# Study on the Compressive Properties of Magnesium Phosphate Cement Mixing with Eco-Friendly Coir Fiber Considering Fiber Length

**DOI:** 10.3390/ma13143194

**Published:** 2020-07-17

**Authors:** Zuqian Jiang, Liwen Zhang, Tao Geng, Yushan Lai, Weile Zheng, Min Huang

**Affiliations:** Department of Civil Engineering, Guangzhou University, Guangzhou 510006, China; zuqianjiang1996@163.com (Z.J.); gengtaoltthl@163.com (T.G.); Ln3maize@163.com (Y.L.); wlzhengaurora@163.com (W.Z.); hm1132628255@163.com (M.H.)

**Keywords:** magnesium phosphate cement, coir fiber, fiber length, compressive properties, energy absorption, micro-morphology

## Abstract

Coir fiber (CF), an eco-friendly and renewable natural fiber, was introduced into magnesium phosphate cement (MPC) mortar to improve its crack resistance. A total of 21 specimens were employed to investigate the failure pattern, compressive strength, stress–strain curve, and energy absorption of MPC with varying CF lengths (0, 5, 10, 15, 20, 25, and 30 mm) after a curing period of 28 days through a static compressive test. The results demonstrated that compressive strength, elastic modulus, and secant modulus decreased with the increase in CF length. However, energy absorption presented a convex curve, which increased to the maximum value (77.0% relative to the value of the specimen without CF) with a CF length of 20 mm and then declined. A series of modern micro-tests were then carried out to analyze the microstructure and composition of specimens to explain the properties microscopically.

## 1. Introduction

Magnesium phosphate cement (MPC), a chemically bonded ceramic [1,2], is a new inorganic cementitious material produced by a neutralization reaction between phosphate and magnesium oxide, and has been widely used in concrete bridge structure reinforcement and road repairment [3,4]. However, its decreased toughness compared to older building materials can result in secondary damage to these reinforced bridges and repaired roads. To overcome this problem, many studies were conducted on incorporating various synthetic fibers, such as steel fiber, glass fiber, and basalt fiber into MPC to improve its toughness. Feng et al. [5] found that the incorporation of micro steel fiber can significantly improve the compressive strength and flexural toughness of MPC composites. Ahmad et al. [6] confirmed that MPC has higher compressive strength and superior ductility when basalt fiber is involved in MPC mortars. Fang et al. [7] reported that glass fiber can effectively prevent the cracking of MPC. However, the application of these synthetic fibers usually results in excessive carbon dioxide emissions and energy consumption during their production and waste treatment, which contribute to the creation and severity a series of environmental issues, such as global warming [8]. An eco-friendly material instead of synthetic fibers must be found to improve these drawbacks of MPC.

Compared with synthetic fibers, coir fiber (CF), as a natural plant fiber, has the advantages of being fully biodegradable, abundantly available, renewable, and environmentally friendly [9,10]. In addition to these remarkable eco-friendly characteristics, CF has remarkable toughness among natural fibers [11,12,13], showing a high potential to effectively improve the toughness and mechanical properties of cement composites. As Thanushan et al. [14] reported, CF can improve the residual strength, ductility, and energy absorption of earth cement blocks. Li et al. [15] found that cementitious composites reinforced by CF have better flexural strength, higher energy absorption, and increased ductility. These cementitious composites are also lighter than other conventional cementitious materials. Thus, CF is thought to have the ability to improve the drawbacks of MPC because it significantly improves other cement composites. As existing studies mostly focused on the effect of other fibers on MPC or the effect of CF on common concrete, a study on MPC reinforced by CF is needed to understand the mechanical properties of this new cement-based composite material for its application in practical works.

In this study, a compressive test was conducted to clarify the effect of CF length on the compressive properties of MPC. A total of 21 specimens were employed in the test to investigate the failure patterns, compressive strength, stress–strain behavior, and energy absorption of MPC with different CF lengths. Modern micro-testing techniques, such as scanning electron microscope (SEM) and energy dispersive X-ray detector (EDX), were adopted to analyze the microstructure and composition of specimens to explain the properties microscopically.

## 2. Experimental Program

### 2.1. Raw Materials

The MPC binder employed in this test was composed of dead-burned magnesium oxide (MgO), potassium dihydrogen phosphate (KH_2_PO_4_), and fly ash (FA), at the KH_2_PO_4_–MgO mass ratio of 0.68, the FA–MgO mass ratio of 0.25, and the water–MPC binder mass ratio of 0.15. Dead-burned MgO powder (calcined at >1500 °C for 6 h) with a specific surface area of 228 m^2^/kg, a density of 2650 kg/m^3^, and an average particle size of 45 µm, was supplied by Zhengyang Casting Material Company (Xinmi, China). Its chemical composition is shown in Table 1. American Society of Testing Materials (ASTM) C618 F class FA was provided by Hengnuo Co. Ltd. (Gongyi, China). Its chemical composition is listed in Table 2. Industrial-grade KH_2_PO_4_, having a purity of 99.5% and an average particle size of 350 µm, was provided by Jiang Hua Chemical Glass Instrument Co. Ltd. of Nanjing, Jiangsu, China. In addition, borax (Na_2_B_4_O_7_·10H_2_O), used as a retarder, with a mass of 10% MgO, a purity of 99.5%, and an average particle size of 350 µm, was supplied by Jiang Hua Chemical Glass Instrument Co. Ltd. of Nanjing, Jiangsu, China. The mixing proportion of these materials is shown in Table 3.

The CF (Jiagaocheng import and Export Trade Co. Ltd., Shangrao, Jiangxi, China), came from Sri Lanka, and had a diameter range of 150–350 µm and a density of 1200 kg/m^3^. Before the CF was mixed with the MPC mortar, it was boiled, dried, and cut into different lengths. Six CF lengths (5, 10, 15, 20, 25, and 30 mm) were used to investigate the effects of CF length on the compressive properties of MPC. The detailed physical and mechanical properties of CF are shown in Table 4.

To prepare the specimens, KH_2_PO_4_, Na_2_B_4_O_7_·10H_2_O, and FA dry powder were first put into a shaft mixer and stirred uniformly for about 30 s. Second, MgO was added into the mixture and stirred continuously for another 30 s. Then, water was poured into the mixture and stirred for 1 min until MPC mortar was produced. After, CF of different fiber lengths was distributed into the MPC mortar and mixed quickly for 30 s. Finally, the well-mixed mortar was pumped into molds and demolded 1 h later to be cured in a room with approximately relative humidity (RH) of 45 ± 5% at 2 ± 3 °C for 28 days. 

### 2.2. Specimens and Test Setup

**Figure 1:**
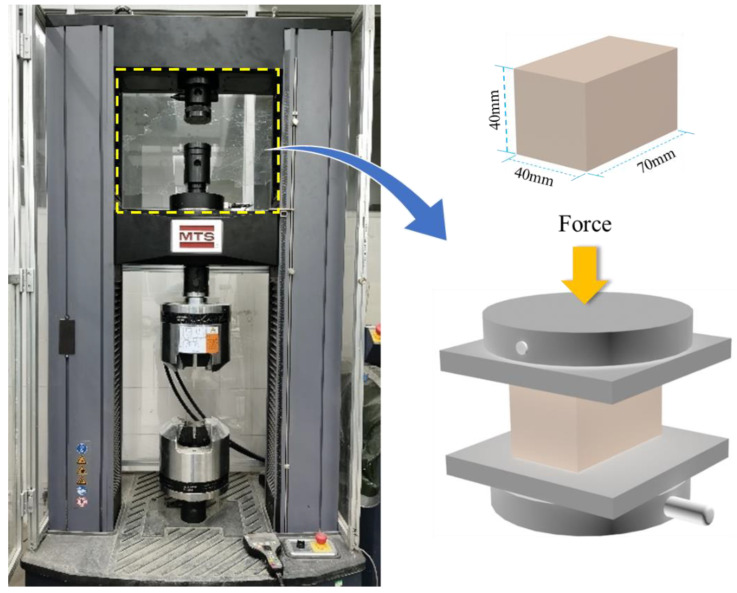
Test setup.

In total, 21 specimens were employed in the test, each with dimensions of 40 × 70 × 40 mm, as shown in Figure 1. The specimens were divided into seven groups according to CF length. One of the groups was designated as the control specimen, which is without CF. Each specimen was labeled test method-CF length-specimen number. For example, SC-CFL10-2 represented the second specimen with 10 mm long CF, which was used to investigate compressive performance. Table 4 offers detailed parameters of all specimens.

A static compression test was carried out using an MTS-E45.305 electronic universal testing machine in reference to the ASTM C109, as shown in Figure 1. First, specimens were preloaded at the rate of 1.0 mm/min until the loading force reached 0.1 kN. Then, loading started at the rate of 2.0 mm/min. The load–displacement (L–D) curve data were recorded in a computer by sensors installed at the load cell. When the loading force dropped to 50% of the peak load, the test was stopped, and the specimen was considered to be a failure. The compressive strength of specimens was calculated by Equation (1) according to ASTM C109-16 [19]:

(1) $$f_{\mathrm{cu}}=\frac{P_{u}}{A}$$

where *f_cu_* is the compressive strength (MPa), *P_u_* is the maximum load (kN), and *A* is the area under compression (*A* = 2800 mm^2^). The elastic modulus of specimens was calculated using Equation (2) in accordance with ASTM C469-14 [20]:

(2) $$E=\frac{\sigma_{2}-\sigma_{1}}{\varepsilon_{2}-\varepsilon_{1}}$$

where *E* is the elastic modulus (GPa), *σ*_1_ is the stress at *ɛ*_1_ = 0.5 uɛ (MPa), *σ*_2_ is the stress increased to 40% of peak stress (MPa), and *ɛ*_2_ is the longitudinal strain corresponding to stress *σ*_2_. For specimen failure, a high-definition camera was used to capture the failure pattern. Samples were also collected from broken specimens, undergoing analyses by SEM, EDX, and X-ray diffraction (XRD) to investigate specimens’ micro-morphology and hydration products to clarify the mechanisms of CF behavior.

## 3. Results and Discussion

### 3.1. Failure Patterns

**Figure 2:**
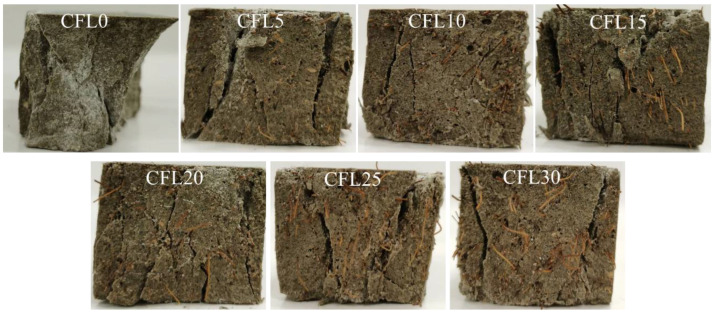
Failure patterns.

**Figure 3:**
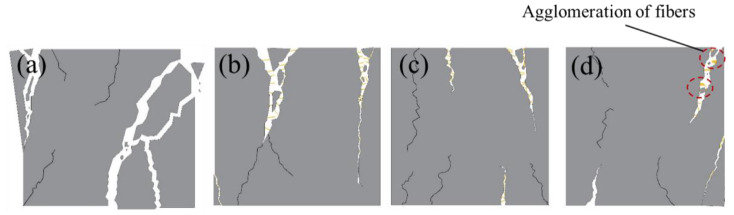
Failure patterns type of the specimen: (**a**) Type I, (**b**) Type II, (**c**) Type III, and (**d**) Type IV.

The failure patterns (Figure 2) from the compression test were divided into four types as displayed in Figure 3. Type I (Figure 3a) was a typical brittle failure, in which specimens failed almost instantaneously when cracks occurred. The MPC matrix broke into pieces after the loading reached the maximum value. Type II (Figure 3b) was similar to Type I, but separated portions did not fall off from the specimens due to the connection of CF in the cracks. Compared with Types I and II, Type III displayed an initial signal of ductile failure. As in Figure 3c, specimens still had a resistance to load after the loading force reached its peak value. With the loading process, cracks were observed more gradually in specimens, and often smaller in width than cracks in Types I and II. Specimens were not penetrated by cracks because CF effectively restricted the development of these cracks. Figure 3d depicts the final type of failure pattern, Type IV, which showed a similar mode to Type III, except that some CF intertwisted or agglomerated in cracks.

Failure patterns of each group are summarized in Figure 2. Type I patterns were found in group CFL0, in which nothing was observed in the initial stage of loading. However, when loading force was close to its peak value, an obvious crack occurred in the specimens and rapidly extended after the peak load until specimens were penetrated. Then, specimens failed instantaneously with a harsh rock-fissuring sound. Group CFL5 presented the Type II failure. It performed like CFL0 in the test process, but did not break up into separated pieces due to CF connecting these pieces together. With CF length increasing, specimens displayed Type III failure patterns. CFL10, CFL15, and CFL20 cracked slowly with frequent sounds of CF rupture and pulled out CF when loading exceeded the peak load. These specimens were greatly improved in axial deformation compared with the specimens that incorporated CF shorter than 10 mm. When CF length exceeded 20 mm, i.e., CFL25 and CFL30, specimens showed the Type IV failure pattern. In this type, some agglomerated CF was observed in cracks due to the overlong CF, resulting in poor fluidity of MPC mortar and inhomogeneous distribution of CF. This meant that no CF was found in some cracks, although specimens still showed greater ductility.

### 3.2. Stress–Strain Behavior

**Figure 4:**
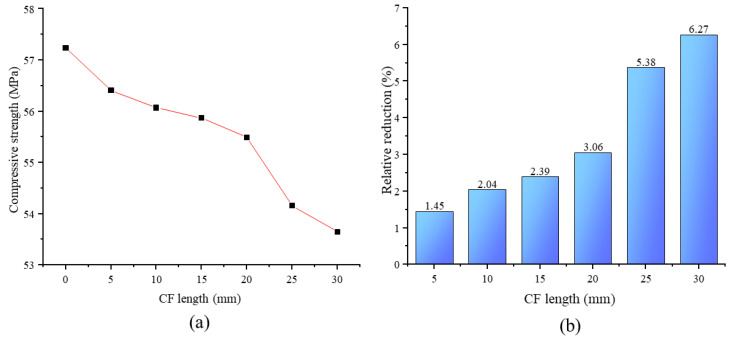
Compressive behavior: (**a**) Compressive strength and (**b**) relative reduction.

Figure 4a displays the average compressive strength (ACS) of specimens. We observed that ACS gradually decreased as CF length increased. When CF length was 30 mm, ACS declined to 53.65 MPa, decreasing by 6.27% relative to the value of specimens without CF (57.24 MPa), as shown in Figure 4b.

**Figure 5:**
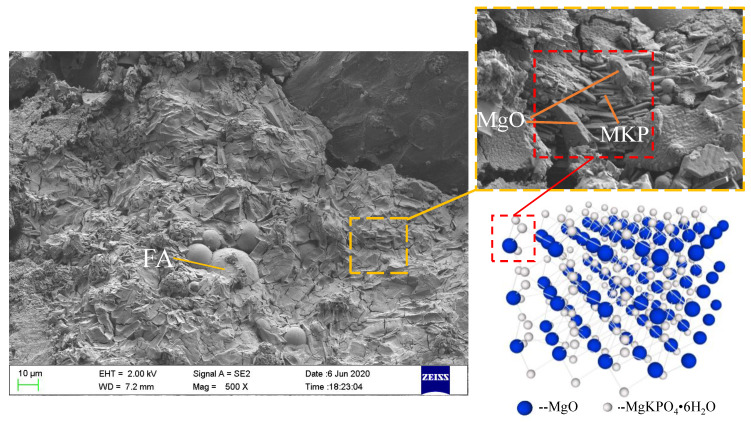
Fracture surface micro-morphology and structure of Magnesium Phosphate Cement (MPC).

**Figure 6:**
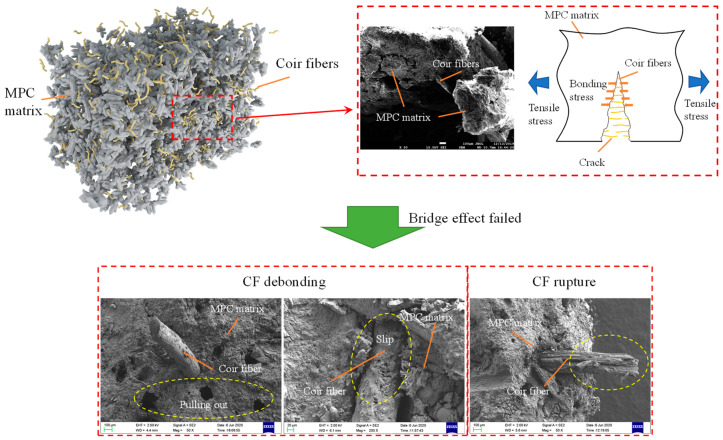
Scanning Electron Microscope (SEM) images and sketches showing the bridge effect during crack propagation.

**Figure 7:**
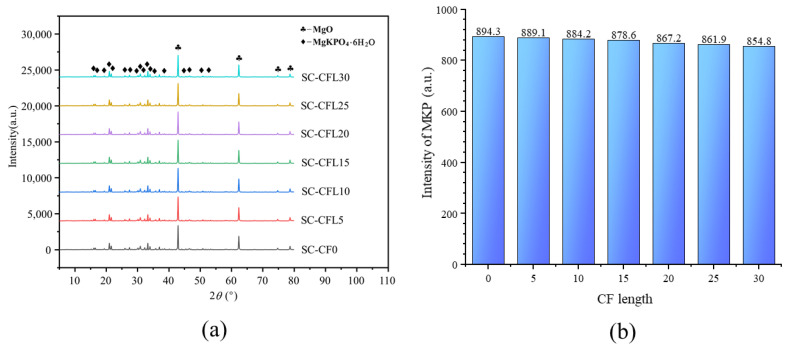
X-ray diffraction (XRD) results for specimens: (**a**) XRD results and (**b**) intensity of MgKPO_4_·6H_2_O (MKP).

**Figure 8:**
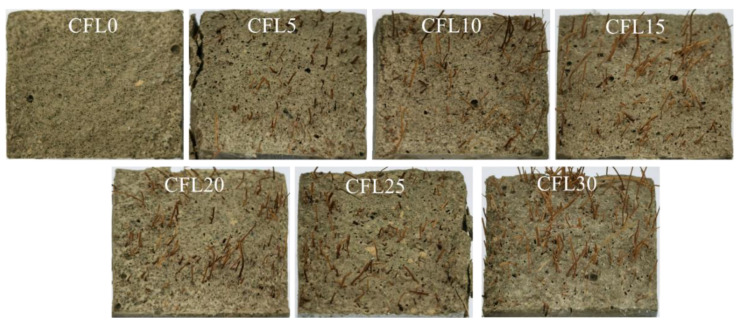
Fracture surface morphology.

This effect of CF length on MPC compressive strength could be explained by the following reasons: In MPC, the main hydration product is a compound called potassium phosphate magnesium (MgKPO_4_·6H_2_O, MKP), also known as K-struvite, which surrounds the unreacted MgO to form a crystal product, i.e., the dominant component providing the mechanical strength of MPC [21]. Figure 5 presents its micro-morphology through SEM and micro-morphological schematics. Then, as shown in Figure 5, the MPC matrix exhibited a resistance to external compression with the help of the space network constituted by these interconnecting crystal products. Although adding CF can restrict the expansion of cracks in the MPC matrix by enhancing the connections of these crystal products through the bridge effect, as shown in Figure 6, it is of little use in improving the compressive strength due to the negligible width of the cracks before the peak load. This phenomenon is also reported in other studies about fibers [14,22]. Adding CF can also lead to the decline in MKP. As shown in Figure 7, the maximum intensity of MKP decreased from 894.3 to 854.8 a.u. when CF length increased from 0 to 30 mm, allowing the MPC to lose a certain compressive strength. Additionally, the increase of CF length reduced CF amount due to the constant proportion of CF in specimens. As a result, the CF per unit volume in the MPC matrix decreased with increased CF length, leading not only to decreased effectiveness of CF, but to nonuniform distribution, which lowered the compactness of the MPC matrix and led to the agglomeration of overlong CF. Figure 8 offers the fracture surface of specimens, in which the aperture and porosity of specimens both enlarge with the increase of CF length. These phenomena had an adverse effect on the mechanical properties of the MPC, resulting in the decrease in ACS.

**Figure 9:**
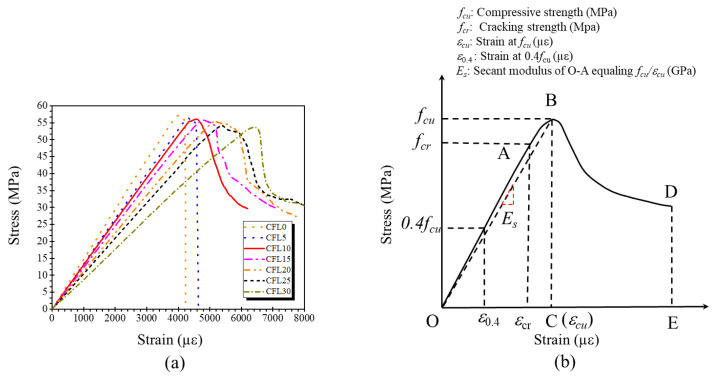
(**a**) Stress–strain curve and (**b**) curve configuration.

Figure 9a depicts the stress–strain curves of specimens in three stages: an approximate elastic stage (O–A), a crack’s stable propagation stage (A–B), and a softening descending stage (B–C, B–D), as described in Figure 9b.

**Figure 10:**
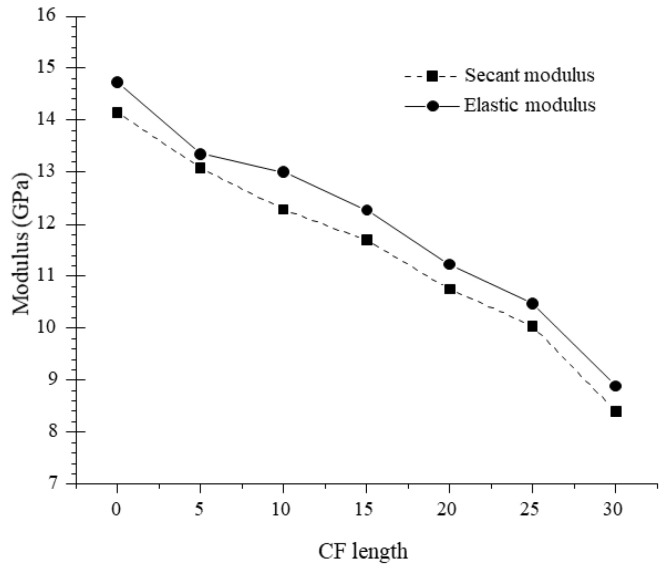
Elastic and secant modulus.

The elastic stage (O–A) can be considered as an approximately linear ascending stage, in which all specimens displayed a similar stress–strain behavior under compression. However, the elastic modulus (*E*) decreased from 14.74 to 8.89 GPa as CF length increased from 0 to 30 mm, shown in Figure 10. The result is attributed to the low elastic modulus of CF which will compromise the stiffness of MPC according to the complex material theory [23]. As described in Figure 8, the compactness of specimens decreased as CF length increased due to the increase of porosity, which also resulted in the decline of *E*. When stress increased to point A as the load increased, some micro-cracks occurred in the matrix due to the local stresses at the cracks exceeding the matrix’s crack-resistance. However, the matrix still kept its capacity due to the cohesion in these cracks, although its elastic modulus degraded gradually as the load increased. In this stage (A–B) for the specimens with CF, the propagation of the cracks was delayed by CF through its bridge effect, which depended on CF’s tensile capacity and its bonding performance with the matrix, as shown in Figure 6. This resistance was more obvious for longer CF. As in Figure 9, the range of A–B and the strain corresponding to the peak stress increased with the increase of CF length. The variation of secant modulus (*E_s_*) could also be used to demonstrate the restrictive effect of CF length on micro-cracks. *E_s_* decreased from 14.14 to 8.40 GPa as CF length increased from 0 to 30 mm (Figure 10).

After point B, the stress–strain behavior entered the softening stage. This stage showed different configurations at different CF lengths. For specimens with CF less than 5 mm, stress is dropping sharply to point C until the specimens failed. When the CF was longer than 10 mm, the stress of specimens decreased slowly to point D, and the specimens still showed notable residual strength and a higher ultimate strain. These phenomena suggest that a shorter CF (less than 5 mm in this study) has a negligible effect on the stress–strain behavior of MPC due to its slight bonding capacity. Figure 7 shows that most CF was pulled out quickly rather than being ruptured for group CFL5, allowing cracks to extend rapidly until the breakdown of the specimens. However, as CF length increased, the bonding capacity improved, so that the CF had sufficient bonding force with the MPC, to allow the CF to stay in the matrix and restrict these expanding cracks through its bridge effect. Those specimens still had an ability to balance load, although their capacity declined with continuous loading. These phenomena also illustrated that the ductility of specimens increased in accordance with the increase of CF length.

### 3.3. Energy Absorption

**Figure 11:**
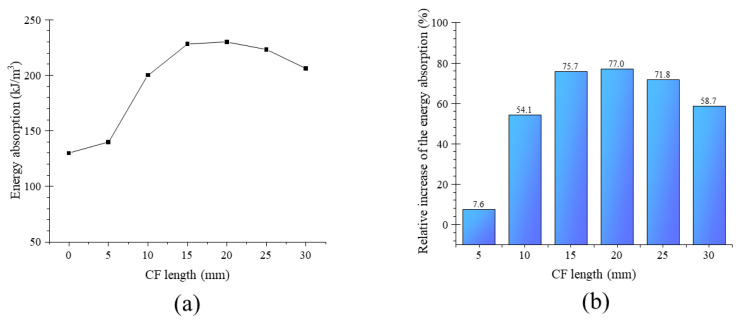
(**a**) Energy absorption and (**b**) relative increase of the energy absorption.

Figure 11 shows the energy absorption of specimens with different CF length, which was defined as the strain energy equaling the area under the stress–strain curve shown in Figure 9b. In this study, the strain at point D was limited to 7000 µε for calculating the area enclosed by stage O–B–D. Figure 11a shows that energy absorption first increased and then decreased with the increase in CF length. When the CF length was 20 mm, energy absorption increased to 230.0 kJ/m^3^, by 77.0% relative to the value of the specimens without CF, shown in Figure 11b. This phenomenon was attributed to the bridge effect of CF, mentioned in Section 3.2. As in Figure 6, the matrix was strengthened after adding CF, benefiting from the connection of the CF for its internal crystal products. If cracks in the matrix developed continuously, the CF eventually slipped out of the matrix, whether through breaking their bonding force or being ruptured, as shown in Figure 6. Whatever resulted, debonding or rupture, energy was consumed and thus would increase with the increase in CF length, due to the increased bonding force.

**Figure 12:**
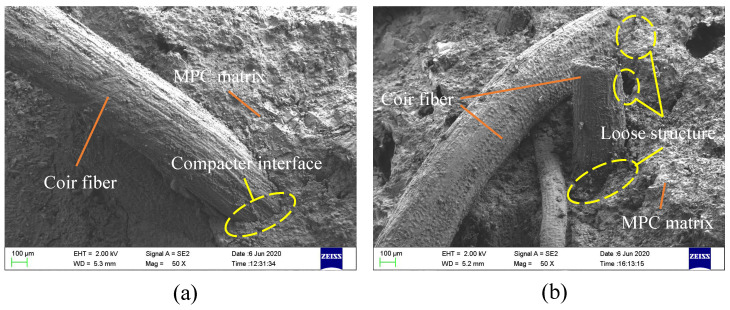
SEM results of boding interface: (**a**) CFL20 and (**b**) CFL30.

However, as described in Section 3.2 (Figure 8), the CF per unit volume decreased with the increase in CF length due to the constant CF proportion in the MPC, leading to the decrease in its effectiveness. Overlong CF (longer than 20 mm in this study) often twined together and weakened mortar fluidity, which had an adverse effect on CF effectiveness, matrix compactness, and CF–MPC bonding performance. CL30 obviously presented a looser structure around CF than CL20 (Figure 12). For that reason, the energy absorption of specimens declined when CF was longer than a threshold (20 mm in this study). Energy absorption decreased from 230.0 to 206.2 kJ/m^3^, and its relative increment dropped from 77.0% to 58.7% as CF length increased from 20 to 30 mm (Figure 10).

## 4. Conclusions

A typical compressive test was performed to study the effect of CF length on the compressive strength, failure pattern, stress–strain curve, and energy absorption of MPC. Our conclusions are summarized as follows:

(1) Adding CF to MPC could effectively increase ductility. When the length of the CF was longer than 10 mm, the failure pattern changed from brittle to ductile.
(2) The addition of CF caused a 6.27% decrease in compressive strength when CF length increased from 0 to 30 mm. The elastic and secant modulus of specimens presented a similar trend as compressive strength, and the plastic properties of MPC showed remarkable improvement.
(3) Adding CF also improves the energy absorption of the MPC, which means increasing the toughness of the MPC. However, this improvement is restricted by CF length. When the length increased to 20 mm, energy absorption reached the maximum value of 230.0 kJ/m^3^ and increased by 77.0% relative to the value of specimens without CF. Then, it decreased in turn as CF length increased continuously.

## Figures and Tables

**Table 1:** Chemical composition of MgO.

Composition	MgO	Al₂O₃	Fe₂O₃	CaO	SiO₂	Loss
**wt %**	96.25	0.29	1.09	1.18	1.16	0.03

**Table 2:** Chemical composition of Fla ash (FA).

Composition	SiO₂	Al₂O₃	Fe₂O₃	CaO	TiO₂	Loss
**wt %**	56.74	24.58	6.55	4.87	1.86	5.4
**ASTM C618** (**%**)	≥70			-	-	≤6

**Table 3:** Coir Fiber (CF) physical and mechanical properties.

Diameter (µm)	Density (kg/m^3^)	Moisture Content (%)	Length (mm)	Tensile Strength (MPa)	Tensile Modulus (GPa)	Elongation at Break (%)
150–350	1200	10–12	5–30	128–157	3.86–5.60	21.2–40.7

**Table 4:** Specimen parameters.

Group	Specimen Number	MPC Binder (Mass Ratio)			B/M ^1^ (%)	W/BM ^2^ (%)	Volume Fraction ^3^ (*V_f_*, %)	Fiber Length (mm)
		MgO	KH_2_PO_4_	Fly Ash				
CFL0	SC-CFL0-1	1.0	0.68	0.25	10	15	3.0	0.0
	SC-CFL0-2							
	SC-CFL0-3							
CFL5	SC-CFL5-1							5.0
	SC-CFL5-2							
	SC-CFL5-3							
CFL10	SC-CFL10-1							10.0
	SC-CFL10-2							
	SC-CFL10-3							
CFL15	SC-CFL15-1							15.0
	SC-CFL15-2							
	SC-CFL15-3							
CFL20	SC-CFL20-1							20.0
	SC-CFL20-2							
	SC-CFL20-3							
CFL25	SC-CFL25-1							25.0
	SC-CFL25-2							
	SC-CFL25-3							
CFL30	SC-CFL30-1							30.0
	SC-CFL30-2							
	SC-CFL30-3							

^1^ B/M is the mass ratio of borax to MgO; ^2^ W/BM is the mass ratio of water to binder; ^3^ 3.0% volume fraction refers to existing research [16,17,18].

## References

[1] Roy D.M. (1987). New Strong Cement Materials: Chemically Bonded Ceramics. Science.

[2] Abdelrazig B.E.I., Sharp J.H., El-Jazairi B. (1988). The chemical composition of mortars made from magnesia phosphate cement. Cem. Concr. Res..

[3] Qiao F., Chau C.K., Li Z. (2010). Property evaluation of magnesium phosphate cement mortar as patch repair material. Constr. Build. Mater..

[4] Yang Q.B., Zhu B.R., Zhang S.Q. (2000). Properties and applications of magnesia-phosphate cement mortar for rapid repair of concrete. Cem. Concr. Res..

[5] Feng H., Chen G., Gao D. (2018). Mechanical Properties of Steel Fiber-Reinforced Magnesium Phosphate Cement Mortar. Adv. Civ. Eng..

[6] Ahmad M.R., Chen B. (2018). Effect of silica fume and basalt fiber on the mechanical properties and microstructure of magnesium phosphate cement (MPC) mortar. Constr. Build. Mater..

[7] Fang Y., Cui P., Ding Z., Zhu J.X. (2018). Properties of a magnesium phosphate cement-based fire-retardant coating containing glass fiber or glass fiber powder. Constr. Build. Mater..

[8] Ahmad M.R., Chen B., Haque M.A., Shah S.F.A. (2019). Development of a sustainable and innovant hygrothermal bio-composite featuring the enhanced mechanical properties. J. Clean. Prod..

[9] Lecompte T., Perrot A., Subrianto A. (2015). A novel pull-out device used to study the influence of pressure during processing of cement-based material reinforced with coir. Constr. Build. Mater..

[10] Paul A., Thomas S. (1997). Electrical properties of natural-fiber-reinforced low-density polyethylene composites: A comparison with carbon black and glass-fiber-filled low-density polyethylene composites. J. Appl. Polym. Sci..

[11] Ali M., Liu A., Sou H., Chouw N. (2012). Mechanical and dynamic properties of coconut fiber reinforced concrete. Constr. Build. Mater..

[12] Ali M., Li X.Y., Chouw N. (2013). Experimental investigations on bond strength between coconut fiber and concrete. Mater. Des..

[13] Baruah P., Talukdar S. (2007). A comparative study of compressive, flexural, tensile and shear strength of concrete with fibres of different origins. Indian Concr. J..

[14] Thanushan K., Yogananth Y., Sangeeth P., Coonghe J.G., Sathiparan N. (2019). Strength and Durability Characteristics of Coconut Fiber Reinforced Earth Cement Blocks. J. Nat. Fibers.

[15] Li Z.J., Wang L.J., Wang X.G. (2006). Flexural Characteristics of Coir Fiber Reinforced Cementitious Composites. Fiber. Polym..

[16] Sekar A., Kandasamy G. (2018). Optimization of Coconut Fiber in Coconut Shell Concrete and Its Mechanical and Bond Properties. Materials.

[17] Hwang C.L., Tran V.A., Hong J.W. (2016). Effects of short coconut fiber on the mechanical properties, plastic cracking behavior, and impact resistance of cementitious composites. Constr. Build. Mater..

[18] Fang Y., Chen B., Oderji S.Y. (2018). Experimental research on magnesium phosphate cement mortar reinforced by glass fiber. Constr. Build. Mater..

[19] ASTM C109-16 (2016). Standard Test Method for Compressive Strength of Hydraulic Cement Mortars.

[20] ASTM C469-14 (2014). Standard Test Method for Static Modulus of Elasticity and Poisson’s Ratio of Concrete in Compression.

[21] Xu B.W., Lothenbach B., Leemann A., Winnefeld F. (2018). Reaction mechanism of magnesium potassium phosphate cement with high magnesium-to-phosphate ratiod. Cem. Concr. Res..

[22] Estabragh A.R., Ranjbari S., Javadi A.A. (2017). Properties of Clay Soil and Soil Cement Reinforced with Polypropylene Fibers. ACI Mater. J..

[23] Swamy P.A.V.B. (1970). Efficient inference in a random coefficient regression model. Econometrica.

